# Targeting glutamate transporter-1 in neurological diseases

**DOI:** 10.18632/oncotarget.16374

**Published:** 2017-03-18

**Authors:** Jacqueline A. Hubbard, Devin K. Binder

**Affiliations:** Center for Glial-Neuronal Interactions, Division of Biomedical Sciences, School of Medicine, University of California, CA, USA

**Keywords:** EAAT2, GLT1, epilepsy, ALS, Alzheimer's

**Figure 1:**
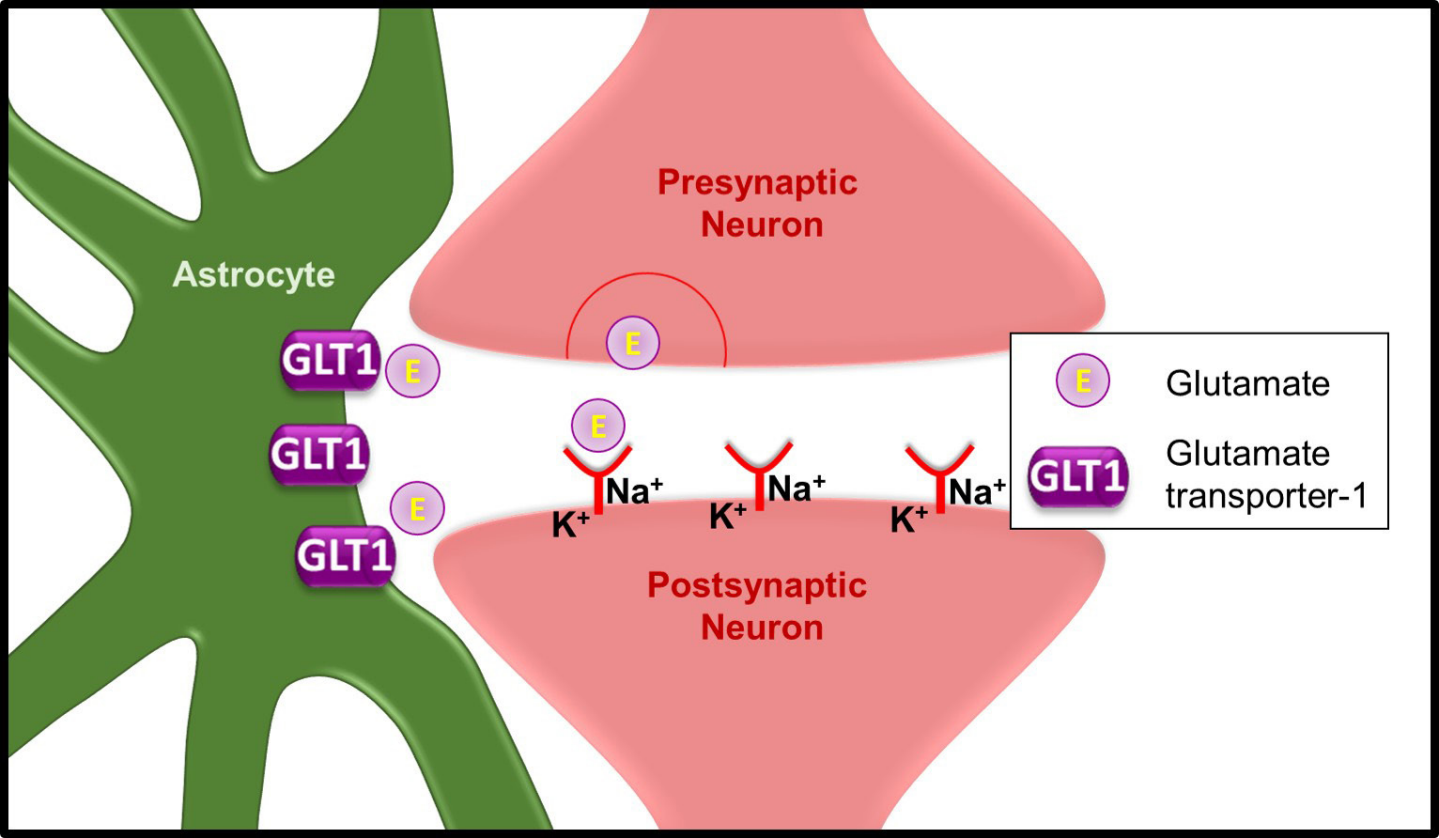
Role of glutamate transporter-1 (GLT1) at the excitatory synapse After glutamate is released from the pre-synaptic neuron, it binds to receptors on the post-synaptic neuron. This allows for the exchange of ions (Na+, K+) and subsequent firing of action potentials. Glutamate then spills out of the synapse and is cleared from the extracellular space by GLT1. Within the astrocyte, glutamate can then be metabolized in a variety of ways.

The accumulation of glutamate in the brain is toxic to neurons and therefore must be tightly regulated. Expressed predominantly on astrocytes, glutamate transporter-1 (GLT1) is responsible for the majority of glutamate clearance from the extracellular space in the forebrain (Figure 1). Due to its importance in glutamate homeostasis and consequently maintaining neuronal health, dysregulation of GLT1 has severe consequences. In fact, GLT1 is found to be downregulated or dysfunctional in several neurological disorders, including epilepsy, amyotrophic lateral sclerosis (ALS), and Alzheimer's disease (AD). Exactly how GLT1 is regulated, however, is an important consideration in each of these diseases.

Epilepsy is comprised of a group of disorders characterized by the unpredictable occurrence of seizures. One of the most common rodent models of epilepsy is the intrahippocampal kainic acid (IHKA) model. Specifically, mice are given a unilateral injection of kainic acid into the dorsal hippocampus. After a prolonged latency period, rodents begin to have spontaneous seizures and are considered epileptic. During that latency period, the brain is transitioning from a healthy one into an epileptic one, a process known as epileptogenesis. Dorsal GLT1 protein expression is downregulated in both hippocampi at time points within the latency period [3]. GLT1 mRNA, on the other hand, is largely unaffected suggesting that GLT1 is regulated at the post-transcriptional level. The fact that GLT1 is downregulated prior to the onset of spontaneous seizures suggests that GLT1 is a key component of the epileptogenic process and therefore is an excellent anti-epileptic therapeutic target. If downregulation of GLT1 could be prevented, it is possible that the development of epilepsy could be prevented altogether.

Amyotrophic lateral sclerosis (ALS) is a progressive neurodegenerative disorder that causes death of motor neurons. Patients exhibit a gradual weakening and wasting of muscles. Originally created in 1994, a common model of ALS is a transgenic mouse line containing a mutation in the Cu2+, Zn2+ superoxide dismutase (SOD-1) enzyme. Specifically, a point mutation at amino acid position 93 (G to A) leads to adult-onset neurodegeneration of motor neurons, progressive motor deficits, and eventual paralysis. Similar to what was observed in the IHKA epilepsy model, focal loss of GLT1 expression was observed before the onset of disease hallmarks (motor neuron degeneration) and continues well after the onset of symptoms [2]. Furthermore, an early study found no change in GLT1 mRNA in the motor cortex of patients with ALS suggesting that GLT1 abnormalities are introduced at the post-translational level [1].

Alzheimer's disease (AD) is also a progressive neurodegenerative disease but it is characterized by memory impairments and progressive dementia. Hallmark of this disease include abnormal deposits of amyloid beta (Aβ) protein and tau, neurofibrillary tangles, and atrophy of affected brain regions. Patients with AD exhibit reduced GLT1 protein levels, but GLT1 mRNA remained unaltered [6]. Therefore, GLT1 is only altered post-transcriptionally. Furthermore, mice lacking one allele for GLT1 who are crossed with mice lacking the amyloid precursor protein (commonly used as an Alzheimer's model) exhibit accelerated cognitive deficits [8]. This suggests that GLT1 contributes to the pathogenesis of AD.

All of these diseases exhibit some form of deteriorating neuronal health. The majority of available drugs to treat them work by acting on neuronal targets. A consequence of this however, is major adverse effects, including cognitive impairment. Therefore, it may be beneficial to seek non-neuronal targets in the brain that can still modulate neuronal health. The regulation of GLT1 is a prime candidate. At least two pharmacological approaches to upregulate GLT1 are available today. First, the β-lactam antibiotic ceftriaxone has been shown to increase GLT1 expression [7] by increasing GLT1 promoter activation [5]. The efficacy of ceftriaxone, however, has been controversial. Second, a novel translational GLT1 activator, LDN/OSU-0212320, has recently been discovered [4]. This compound may be a more promising therapeutic for conditions in which GLT1 is regulated at the post-transcriptional or translational levels. To support this, LDN/OSU-0212320 has already shown therapeutic efficacy in epilepsy [4], ALS [4], and AD [8] models.

Here we have highlighted the role of GLT1 in just a few disorders that affect the CNS. It is crucial to find drugs that can specifically target GLT1 on both the transcriptional and translational level to ensure that optimal treatment is used for every distinct condition. Although only epilepsy, ALS, and AD were discussed here, this concept is applicable to other neurological disorders in which GLT1 is downregulated including schizophrenia and Huntington's disease. Furthermore, in disorders that progress slowly, early treatment with a GLT1 modulator has the potential to prevent the full progression of the disease. Further disease-specific progress in GLT1 therapeutics will come from: (1) understanding of the timing of GLT1 dysregulation relative to disease stage/expression; and (2) precise mechanism of GLT1 protein dysregulation (e.g. translational regulation or post-translational modification leading to altered GLT1 subcellular targeting).
